# Neutrophils in cancer: from biology to therapy

**DOI:** 10.1038/s41423-024-01244-9

**Published:** 2024-12-09

**Authors:** Leo Koenderman, Nienke Vrisekoop

**Affiliations:** Dept. Respiratory Medicine and Center for Translational Immunology, University Medical Center Utrecht, Utrecht, The Netherlands

**Keywords:** neutrophil, immune suppression, cancer, tumor-associated neutrophils, confused immune response, myeloid derived suppressor cells, Immunosurveillance, Cancer microenvironment, Granulocytes

## Abstract

The view of neutrophils has shifted from simple phagocytic cells, whose main function is to kill pathogens, to very complex cells that are also involved in immune regulation and tissue repair. These cells are essential for maintaining and regaining tissue homeostasis. Neutrophils can be viewed as double-edged swords in a range of situations. The potent killing machinery necessary for immune responses to pathogens can easily lead to collateral damage to host tissues when inappropriately controlled. Furthermore, some subtypes of neutrophils are potent pathogen killers, whereas others are immunosuppressive or can aid in tissue healing. Finally, in tumor immunology, many examples of both protumorigenic and antitumorigenic properties of neutrophils have been described. This has important consequences for cancer therapy, as targeting neutrophils can lead to either suppressed or stimulated antitumor responses. This review will discuss the current knowledge regarding the pro- and antitumorigenic roles of neutrophils, leading to the concept of a confused state of neutrophil-driven pro-/antitumor responses.

## Introduction

### Neutrophils as double-edged swords in the effector phase of the immune response

The effector cells of the immune system have two faces as their beneficial functions are carried out via tissue damaging mechanisms [1]. The toxic effector functions associated with antimicrobial defense easily cause collateral damage to the host tissue, and in the case of hyperactivation, the increased damage outweighs the benefits [2]. In addition to toxic effector neutrophils, other neutrophil phenotypes appear during wound healing, which aids in angiogenesis and tissue repair [3]. Tumors have also been described as wounds that never heal, and they can hijack the normal wound healing response for their own growth and spread [4]. A third two-sided role of neutrophils is found in tumor immunology, as the cells can be pro- and antitumorigenic depending on the phenotype of the neutrophil, the timing and the tumor type.

Most mechanistic studies on the role of neutrophils in tumorigenesis are performed with mouse models. Unfortunately, the neutrophil compartment in mice is very different with respect to neutrophil functions. It is beyond the scope of this article to review them all [5, 6]. However, some differences, such as the number of cells, kinetics, receptors, phenotypes and nuclear morphology, are crucial for the translation of murine data related to neutrophil-mediated tumor immunology to humans. First, the number of neutrophils in the peripheral murine blood is much lower (≈10% in spf mice vs ≈60% in humans) [5, 6]. The differentiation time from the last division in the bone marrow and exit to the blood is <1 day in mice and 6 days in humans [7, 8]. Although the lifespan of human neutrophils is debated, as discussed later in this review, it is generally accepted that human neutrophils live longer than their murine counterparts do [7, 9]. This has important consequences, as the inherently slower neutrophil system in humans leaves more time for these cells to engage in immune regulation [7]. Murine neutrophils also have completely different repertoires of Fc-receptors, as illustrated by different FcγRs [10] and the absence of FcaR [11, 12] on murine cells. Finally, murine neutrophils are smaller and have a typical donut-shaped nucleus, whereas human cells contain nuclei with multiple segments [5]. In conclusion, great care should be taken when translating murine data to the human situation. Therefore, our review is focused on human data. Nevertheless, mouse studies can provide mechanistic insights for phenomena found in humans. In the event that substantiating data originate from mouse experiments, this will be indicated.

To understand the complex role(s) of neutrophils in tumor biology, it is essential to first discuss the function of the neutrophil compartment in health/immune homeostasis. Several basic mechanisms are described: (1) the lifespan of neutrophils, as this determines how long tumor cells can be influenced by individual neutrophils; (2) the presence of different phenotypes, as their functional differences determine whether cells are involved in pro- and/or antitumorigenic responses; (3) the activation of different functions determines the overall response toward microorganisms and tumor cells; and (4) the role of tissue cues and plasticity, through which cells adapt their functionality, determines the overall response to tumor cells. The situation of homeostasis and inflammation will be discussed first, followed by the role of neutrophils in tumor progression. Finally, we discuss the implications of the potential role of neutrophil biology in cancer therapy. Recently, several authoritative reviews on the role of neutrophils in antitumor responses have been published. These reviews focused on the identification of novel neutrophil subtypes via unbiassed techniques such as single-cell RNA sequencing (scRNA-seq) [13, 14] followed by analysis via complex nonintuitive algorithms, which are carried out mainly in mice. Unfortunately, the newly identified clusters await functional characterization. The current review focuses more on data obtained via functional characterization on the basis of data acquired via hypothesis-driven studies. These studies highlight the confused neutrophil response in tumor immunology, which involves its normal armamentarium toward tumor and host cells rather than toward microorganisms (Table 1). Therefore, it is important to describe these adequate mechanisms first in health followed by a description of the ‘confused’ response in the tumor microenvironment (TME).

**Table 1:** ‘Double edged sword' mechanisms underlying neutrophil responses in health and in tumor immunology

	Anti-microbe immunology: adequate neutrophil response	Anti-tumor immunology: confused neutrophil response	References
Induction of immune suppressive neutrophils/ MDSC	Prevention of hyper-inflammation by immune regulation	**Suppression of anti-tumor responses**	[106, 111–118], [128, 159–169], [171]
Homing of neutrophils towards tissue	Homing of neutrophils towards tissue location with microbes	**Formation of the pre-metastatic niche, tumor cell dissemination**	[22–24, 62, 94], [207–210], [211–223]
Antibody dependent cellular cytotoxicity (ADCC)	Killing of pathogens with minimal damage to healthy tissue	Killing of (tumor) tissue with possibly suppressed anti-bactericidal function	[11, 12, 240–244], [246–258]
Complement dependent cytotoxicity (CDC)	Killing of pathogens with minimal damage to healthy tissue	Killing of tumor tissue with possibly suppressed anti-bactericidal function	[246, 253, 259–262]
Trogocytosis	Killing of large targets such as fungi and parasites	Killing of opsonized large tumor cells	[236, 237]
Apoptosis	Control of the balance of the neutrophil response	**Increased survival of neutrophils leading to collateral damage of host tissue**	[48–50, 68–70], [123, 124, 281]
Netosis	Bactericidal action upon death of the neutrophil	**Pro-tumorigenic mechanism by proinflammatory signals**	[70, 95, 96, 224], [226]
Phagocytosis and killing	Elimination of micro-organsims	**Collateral damage to host tissue facilitating tumorigenesis**	[71–74, 137–140], [191–193, 204], [218–220, 224], [226]
Antigen-presentation	Presenting microbe antigens to T cells in lymph nodes	Presenting tumor antigens to T cells in lymph nodes	[272–275]
Inducing cell proliferation	Wound healing	**Increased tumor cell proliferation**	[3, 4, 62, 187–189, 192,193]
Angiogenesis	Wound healing	**Dissemination of tumor cells and growth of tumors**	[3, 4, 62, 185, 186], [196–200]
Inducing EMT and cell motility	Wound healing	**Dissemination of tumor cells**	[3, 4, 202–206]

The balanced neutrophil response that allows sufficient cytotoxicity against invading micro-organisms with limited collateral tissue damage becomes deviated in the tumor micro-environment (in bold). This leads to both beneficial anti-tumor tissue responses as well as pathological suppression of innate immune responses. Dealing with this confused neutrophil response is very difficult as the balanced immune response necessary for immune homeostasis has now evolved into two extremes: immune suppression and cytotoxicity that can be both pathological as well as beneficial. This calls for new anti-neutrophil therapy targeted at the tumor environment, as successful anti-tumor responses need a mirror like response: cytotoxic to (tumor) diseased tissue without collateral suppression of anti-microbial and/or immunoregulatory responses

## Neutrophils in health

### The neutrophil compartment

Phagocytes (cells that can take up/phagocytose small biotic and abiotic targets) occurred in the evolutionary tree in the kingdom of animalia approximately 800 million years ago [15]. To date, these cells are found in multiple species ranging from very simple, such as corals [16], to highly complex, such as primates [17]. During evolution, phagocytes found in complex species have evolved into multiple specialized cell types, such as monocytes, macrophages and granulocytes [18]. In these cell types, several mechanisms have been developed that enable these cells to kill or at least contain (inhibit intracellular growth without killing) the phagocytosed targets (see also the following section). Different phagocytes have different specialized characteristics/functions and are therefore found at different tissue locations [18].

Neutrophils are granulocytes that are involved in the defense against small targets (<10 μm). They are phagocytes that are normally present in the peripheral blood in large amounts (≈20 × 10^9^ per person of 70 kg) [19] but are much more abundant in the bone marrow (≈300 × 10^9^ per person of 70 kg) [19]. In mice, neutrophils are also found in the liver and spleen [20]. Neutrophils are typically found in low numbers in other healthy tissues but are mobilized in response to infectious and/or inflammatory cues in the event that the mononuclear phagocyte compartment is not able to address the invading pathogen(s) [21]. The route of neutrophils toward the inflammatory locus involves inflammatory mediators that are produced by inflammatory and bystander cells. Blood neutrophils in the vicinity of an infection/inflammation locus recognize activated endothelial cells that start expressing rolling receptors [22] as well as chemokines [23]. The rolling receptors are involved in slowing down the cells in the vasculature, and chemokines originating from bystander cells lead to the activation of neutrophil integrins [22], which in turn facilitate firm adhesion and transendothelial migration [24]. Once in the tissue, neutrophils find their way to the inflammatory/infectious locus via chemotactic cues liberated by pathogens (e.g., formyl peptides) and locally produced chemotaxins by both inflammatory and bystander cells. At the site of infection, small pathogens are phagocytosed and killed by a variety of killing mechanisms.

### Life cycle of a neutrophil

#### Origin of neutrophils

The neutrophil compartment primarily differentiates in the bone marrow before being released as mature cells into the peripheral blood [25–27]. Like all blood cells, neutrophils originate from pluripotent stem cells in the bone marrow. The first steps in the differentiation and proliferation of these stem cells lead to the choice between the myeloid and lymphoid lineages [28]. This does not seem to be an “irrevocable” choice, as at least in mice, early cells can transdifferentiate between these two main differentiation routes [29]. Hereafter, the cells differentiate into progenitors that are committed to, e.g., the neutrophil lineage. Importantly, it is still debated which progenitor is the first cell committed to the neutrophil lineage [27, 30, 31], but this fundamental issue is not important for the understanding of the current review. After lineage commitment, the cells proliferate and concomitantly differentiate through different stages into neutrophil myeloblasts, pro-myelocytes and myelocytes and are referred to as the mitotic pool [28, 32]. Thereafter, the cells stop proliferating and differentiate into meta-myelocytes, banded neutrophils and mature neutrophils [32]. These cells belong to the postmitotic pool.

#### Pool sizes

##### The mitotic pool of proliferative progenitors

Despite the multitude of studies focusing on the neutrophil compartment in the bone marrow, little if any consensus is present on the pool sizes of the different neutrophil progenitors. The pool sizes of early progenitors, multipotential progenitors and myeloblasts are clearly small, and a large proportion of the more undifferentiated progenitor cells are not in the cycle [32]. Therefore, these cells are important in maintaining the compartment but are not the main source of neutrophils, as the ‘flux’ through these cells is limited [9, 32, 33]. The largest population of neutrophil progenitors that highly proliferate are promyelocytes [19, 34]. As such, these promyelocytes are the main producers of cells of the mature neutrophil compartment, and their kinetics are rate limiting [9]. Unfortunately, the size of the pool of promyelocytes is currently unclear. Multiple studies applying different technologies have attempted to estimate this number, and the data range is approximately twofold from 44 × 10^9^ cells/individual to 72 × 10^9^ cells [9, 25, 34]. There are multiple reasons for this difference. An important issue is the source of the bone marrow, as this tissue consists of a fluid phase and a bone niche. Many studies use bone marrow aspirates, which might overrepresent cells from the fluid phase and be relatively deficient in cells present in the bone niche [35]. Another way of collecting bone marrow is through trephine biopsies [36], but these biopsies are difficult to quantify and extrapolate. Few studies have compared the two methods, but those that have been published conclude that the percentages of different progenitors in aspirates and trephine biopsies are similar [36]. Another complicating factor in the interpretation of bone marrow aspirates is hemodilution of the aspirate by cells originating from the peripheral blood [37]. This does not affect the total number of promyelocytes in the bone marrow but can influence the fraction of these cells in the aspirate, as a significant number of mature neutrophils can originate from the peripheral blood during the procedure. As the promyelocyte fraction is rate limiting in the production of the neutrophil compartment, the rate of proliferation of promyelocytes is key in understanding the regulation of the neutrophil compartment. Myelocytes are traditionally thought to also proliferate and be part of the postmitotic pool, but a recent study has challenged this dogma [32].

##### The postmitotic pool of nonproliferative progenitors

Next, the cells lose their capacity to divide and enter a differentiation/maturation program via meta-myelocytes and banded cells toward mature neutrophils that are mobilized to peripheral blood. For reasons similar to those for promyelocytes, only a few studies have determined the absolute pool sizes of postmitotic progenitors [28, 38]. Most studies describe percentages of progenitors in aspirates rather than absolute numbers and show that the pool sizes of myelocytes, metamyelocytes and banded cells are similar [19, 25, 32]. The issue of the pool size of the last step, mature neutrophils, is unresolved. The estimates in the literature range from 99 × 10^9^ cells to 415 × 10^9^ cells in the total bone marrow [19, 25, 34, 39–41]. Together, a twofold difference in the estimated promyelocyte pool and a fourfold difference in the estimated mature neutrophil pool result in an eightfold range of estimated production. This lack of consensus precludes a detailed understanding of the neutrophil compartment.

#### Basic kinetics

In addition to determining pool sizes, measuring the kinetics and tissue distribution can help clarify the potential roles and limitations of mature neutrophils. In particular, the relative longevity (t½ of multiple days) of neutrophils supports their role in immune regulation and in tumor immunology. However, in the late fifties/early sixties of the last century, sizeable literature led to textbook knowledge that human neutrophils are short-lived in the peripheral blood (t½ of 7 h) [34, 42–44]. All these studies used essentially the same experimental approach of labeling neutrophils in vivo or in vitro with radioactive diisopropyl fluorophosphate (DFP), which irreversibly binds to neutrophil elastase [45] and followed the decay in radioactivity in blood neutrophils in time. The implicit underlying hypothesis was that the half-life in peripheral blood equals the death of the cells in the neutrophil compartment. However, an alternative hypothesis of dilution of cells in a large population of neutrophils outside the peripheral blood (e.g., bone marrow) can explain the data at least equally well [9].

More recent analyses applying stable isotope labeling in humans also did not support such a short half-life [7] but did not lead to a consensus on the lifetime of a mature neutrophil [8], leading to estimates between 1 and 5.6 days. The reason for these contradictory findings lies in the uncertainties with respect to pool sizes and the unknown kinetics of promyelocytes [46, 47, 10].

#### Tissue distribution

Apart from pool size and kinetics, the tissue distribution of the neutrophil compartment in homeostasis has not been well described. Here, two mutually exclusive hypotheses exist: the ‘tissue surveillance’ model and the ‘peripheral blood surveillance’ model.

The first hypothesis assumes that neutrophils home randomly to tissues and engage in immune surveillance even in the absence of inflammatory/infectious cues. This hypothesis supports the idea that once neutrophils enter tissues, they are cleared by tissue macrophages upon completion of their lifetime, a process generally referred to as efferocytosis [48–50]. Alternatively, the cells may move back from the tissue via reverse migration into the peripheral blood and re-enter the bone marrow for clearance by local macrophages, such as those found in nonextravasated neutrophils [51–54]. Evidence for reverse migration comes from zebra fish, where this has been shown in vivo [51, 55]. However, for humans, little evidence exists for the reverse migration of neutrophils [52, 56], with the main problem being the difficulty of finding neutrophils in healthy tissues in homeostasis.

The second hypothesis assumes that neutrophils only home to tissues upon exposure to infectious/inflammatory cues. In homeostasis, these cells do not migrate over the endothelium and reverse migrate back to the blood, but the cells show patrolling behavior at the vessel wall [57–59]. In the absence of inflammatory cues during their lifetime, aging cells start to express receptors such as the chemokine receptor CXCR4, which are instrumental for aged cells to return to the bone, where they are cleared by bone marrow macrophages. This type of circulatory behavior implies that healthy tissue is devoid of neutrophils, which fits with the lack of data showing appreciable amounts of neutrophils in tissues other than the bone marrow, liver and spleen. Importantly, these hypotheses have been tested mainly in murine models. Data on CXCR4-mediated homing of neutrophils in humans are scarce [60].

Neutrophil differentiation can also occur at secondary sites in a process referred to as extramedullary hematopoiesis. In addition to bone marrow, neutrophils are also found, e.g., in the spleen and liver of mice during homeostasis [20]. An important hypothesis is that these organs are a place for extramedullary granulopoiesis [53, 61]. The relative importance of extramedullary granulopoiesis in mice is not completely clear, and human data are currently lacking [54]. Whether neutrophils home to tissues during immune responses that are out of control is not well understood. Importantly, the neutrophil compartment is heterogeneous, and different phenotypes have different functions.

#### Tissue repair

In addition to being a mobilizable pool of effector cells that deal with invading pathogens, neutrophils have also been described as important in tissue repair [62]. This critical function has been relatively poorly investigated, but fracture hematomas are a clear example of how neutrophils can engage in repair mechanisms [63–65]. When long bones such as the femur break, a fracture hematoma quickly forms. This hematoma is critical in bone repair, as ‘washing’ this tissue from immune cells impairs bone repair, leading to pseudoarthrosis and nonunion (the absence of interactions between bone fragments) [64]. Neutrophils constitute the majority of white blood cells that are found in the hematoma during the first few days after fracture. They contribute to bone repair by synthesizing fibronectin, which functions as an emergency stroma that facilitates and prepares mononuclear cells to arrive and produce bone-specific collagens [64, 66]. This repair response is likely mediated by the production of damage-associated molecular patterns or DAMPs originating from damaged cells and bone structures. This pathway is initiated not only in the case of fractures but also in all cases of tissue injury, including injury caused by tumors. It is tempting to speculate that tumors can hijack neutrophil repair responses to their own benefit [3, 4].

#### Death of neutrophils

The mechanisms of death of neutrophils in homeostasis are very poorly understood in both mice and humans. This poor understanding concerns essentially all aspects of cell death, from the underlying mechanism(s) to the location in the body where clearance of (dead) neutrophils occurs [6, 9]. Whether neutrophils patrol tissues or the circulation (discussed in Section “Tissue distribution”) is one of these uncertainties. The prevailing idea is that aged neutrophils upregulate their CXCR4 receptor in the circulation to facilitate their return to and retention in the bone marrow [67, 68]. In mouse bone marrow, neutrophils are then phagocytosed by bone marrow macrophages via efferocytosis [69, 70]. On the other hand, the possibility that the cells are cleared in distant tissues by local tissue macrophages cannot be ruled out. The relative contribution of both concepts is difficult to prove, as efferocytosis of aged or apoptotic neutrophils is very difficult to detect both in bone marrow and in tissue. To date, the role of neutrophil death in homeostasis has not been resolved. The situation in infection/inflammation is drastically different, as local massive cell death of neutrophils can be observed under these conditions. The green aspect of pus is caused by the liberation of (green) myeloperoxidase from dying neutrophils. The general idea is that dying neutrophils can contribute to the killing of pathogens by forming neutrophil extracellular traps (NETs) and the liberation of toxic constituents from neutrophil granules [70].

### Effector functions

Neutrophils contain a large armamentarium of cytotoxic mechanisms that can be broadly divided into cytotoxic proteins/enzymes, peptides, lipids, low pH, and oxidants [71]. As neutrophils are particularly important in the intracellular killing of pathogens, these mechanisms focus on the killing of pathogens present inside phagolysosomes (the pathogen trapped by the cell after phagocytosis) [72], which minimizes the release of toxic mediators into the area around the cell and thereby limits collateral damage to healthy tissue. The release of toxic mediators outside cells (e.g., by degranulation) occurs when the infection cannot be contained by phagocytosis alone [72]. While the release of toxic mediators facilitates the extracellular killing of pathogens, it also leads to collateral tissue damage [73, 74]. Balanced killing of pathogens is thus essential for preventing overkill resulting in tissue damage.

Important killing mechanisms for both pathogens and tumor cells include the following:

#### Reactive oxygen species (ROS) [75]

Neutrophils contain a membrane-bound NADPH-oxidase (NOX-2) complex that can reduce molecular oxygen to superoxide [76]. In addition to direct cytotoxicity, this oxygen intermediate can be converted to an array of reactive oxygen species [77]. Importantly, cytotoxic hydrogen peroxide (H_2_O_2_) is formed via the action of superoxide dismutase. H_2_O_2_ is subsequently converted to HOCL (bleach) by myeloperoxidase present in primary neutrophil granules [78]. In addition to these three major ROS, other metabolites, such as singlet oxygen and hydroxyl radicals, are formed [79]. The importance of ROS in the killing of microbes is illustrated by the increased occurrence of infections in patients who are deficient in the NADPH-oxidase complex [80]. These chronic granulomatous disease patients need life-long clinical support to prevent bacterial infections [81, 82].

#### Toxic proteins

In addition to myeloperoxidase, several other proteins are present in the granules of neutrophils that contribute to the killing of microbes [83, 84]. Several proteases (e.g., elastase, cathepsin-G, and collagenase) that facilitate both the killing and movement of neutrophils in the matrix are important [85]. In addition to these enzymes, S100 proteins (S100A8/MRP-8, S100A9/MRP-9 and S100A12) are abundantly present in the cytosol [86]. These proteins are released from neutrophils via poorly understood mechanisms. These proteins are not directly cytotoxic but rather engage in Toll-like receptor and receptor for advanced glycation end products (RAGE) signaling pathways in bystander cells [87, 88]. Importantly, a clear synergy is present in the cytotoxic action of both ROS and toxic proteins [89].

#### Cytotoxic peptides

In addition to proteins, neutrophils contain an array of small cytotoxic peptides that directly interact with the membrane of the pathogen [90]. Clear examples include the families of α-defensins [91] and cathelicidins [92]. These peptides are considered to belong to evolutionarily old antimicrobial mechanisms

#### Immune-modulating lipids

The membranes of neutrophils are relatively rich in arachidonic acid, which is the source of the prostaglandins (produced by cyclooxygenase) and leukotrienes (produced by different lipoxygenases) pathways [93]. These lipids are not involved in direct cytotoxicity but are immunomodulatory. For example, leukotriene B_4_ is important because it is an activator of the overall killing of pathogens by inducing neutrophil swarming (induction of increased cellular movement), facilitating contact between neutrophils and their target cells [94].

#### Neutrophil extracellular traps (NETs)

Neutrophils contain a peculiar killing mechanism of pathogens associated with their own death: NETosis [95]. Here, dying neutrophils expel their DNA coated with antimicrobial substances as a net to catch microorganisms in the vicinity of the dying cell. It is still an open question whether NETosis is well regulated or whether it is an all-or-nothing response of dying cells. Importantly, NETosis is considered a proinflammatory process [96] that is associated with pyroptosis and necrosis [97].

### Phenotypes

#### Nomenclature describing the heterogeneous neutrophil compartment

In the last decade, many studies have indicated that the neutrophil compartment is very heterogeneous and characterized by the occurrence of several neutrophil types with specific functions. The terms used to specify these types are phenotype, subtype, and neutrophil type without clearly defining the similarities and differences between these types. The neutrophil field is now increasingly adopting the view that a *neutrophil subtype* is a cell that develops early in differentiation as a separate lineage in the bone marrow, and this process is under the control of specific transcription factors. The only clear example is the finding that homeostatic neutrophils in the bone marrow of mice are under the control of C/EBPα, whereas cells originating from emergency neutropoiesis are driven by C/EBPβ [98]. A *phenotype* occurs after maturation and bone marrow release in response to tissue cues that can lead to a plethora of cellular states and functions [99].

#### Timing of differentiation

Determining which of the neutrophil phenotypes are activation stages of cells derived from a single progenitor source or which subtype has differentiated in parallel with other differentiation paths is crucial. This research question is very difficult to solve in humans, as technology such as genetic fate mapping is very difficult to perform in humans. An interesting study by Grieshaber-Bouyer et al. [100] involved a pseudotime analysis of developing neutrophils in the bone marrow, blood and tissue of mice. This ‘neutrotime’ analysis supports the concept that murine neutrophils differentiate as a single continuum and that phenotypes are all induced by tissue/disease cues outside the bone marrow. To validate the model, they applied early and late neutrotime murine RNAs on a human dataset and presented a similar picture. Unfortunately, the analysis of the human data does not rule out that human cells originate from parallel differentiation paths because there is quite a broad distribution of late neutrotime RNAs in their UMAP plots.

#### Different neutrophil phenotypes

The heterogeneous neutrophil compartment is characterized by cells with different phenotypes, which are characterized by different expression levels of (immune) markers and differences in functionality. Because it is unclear whether these different neutrophil states occurred during development in the bone marrow or later in response to tissue cues, this review adopts the more cautious term “phenotype” when describing the heterogeneity of the neutrophil compartment. The different phenotypes are briefly discussed (for more detailed information, see recent reviews [101–104]).

#### -Normodense and hypodense neutrophils

Neutrophils are normally isolated via density centrifugation because neutrophils (and eosinophils) have a higher buoyant density than mononuclear cells do. It seems that a shift to a lower buoyant density of hypodense neutrophils occurs via the activation of neutrophils in vitro and in vivo and does not necessarily reflect a true subtype originating from the bone marrow. This has recently been reviewed [105].

#### -G-CSF mobilized the banded cells

In healthy donors who are treated with G-CSF for mobilization of progenitor cells (for transplantation purposes), two phenotypes of neutrophils are found in the peripheral blood: a CD10^bright^ population and a CD10^dim^ population [106]. These two populations have different functions but seem to consist of two populations belonging to the same differentiation pathway: CD10^dim^ cells, which are younger cells, and CD10^bright^ cells, which are older cells [107]. This finding fits the hypothesis that G-CSF and inflammation cause a ‘leftward shift’ by mobilizing banded neutrophils and young CD10^dim^ cells from the bone marrow. A complicating factor is that CD10 is also an activation marker upregulated by activation by, e.g., formyl peptides [108].

#### -Cells differentially expressing L-selectin (CD62L) and FcγRIII (CD16)

The abovementioned ‘left shift’ is characterized (apart from low CD10 expression) by a low expression level of CD16 [107]. Low expression of both CD16/CD10 is thus a good indication for recent emigrants from the bone marrow [107]. This ‘left shift’ is a well-known phenomenon in both inflammatory and infectious diseases. Suggestions in the literature that these cells are part of a compensatory response consisting of poorly activatable cells are not true, as these CD16^dim^ banded cells have a superior response in the killing of bacteria such as *S. aureus* [109]. In addition to the mobilization of CD16^dim^ cells, inflammation/infection also induces the occurrence of neutrophils, which are CD62L^dim^/CD11c^bright^ and contain a hypersegmented nucleus, in the blood [110, 111]. These cells are characterized by the poor killing capacity of bacteria but have a greater immunoregulatory function, as they can inhibit the proliferation of T cells [111, 112].

#### -Neutrophilic myeloid-derived suppressor cells (MDSCs)

The concept of immunoregulatory neutrophils has been investigated in detail, and the field has expanded significantly through the identification of myeloid-derived suppressor cells [113]. These cells, which are of monocytic and neutrophilic origin, are able to suppress T-cell proliferation and activation via several mechanisms, including the production of arginase 1 [114, 115], PGE_2_ [116]_,_ reactive oxygen species [111, 112] and transforming growth factor-β1 [117]. Despite the fact that MDSCs were originally described as relatively undifferentiated, later studies showed that neutrophil-derived MDSCs can also be fully differentiated and might resemble CD16^bright^/CD62L^dim^ neutrophils [118]. As these cells play a role in antitumor immune responses, they will be discussed in a separate section in more detail later.

#### -Olfactomedin-4 (OLFM-4)-expressing neutrophils

Clemmensen et al. [119] described two discrete subsets of neutrophils that either express or do not express the intracellular marker OLFM-4. Analysis of functional differences between the two discrete subsets is difficult, as the cell needs to be permeabilized to be assigned to one of the two subtypes. Therefore, little is known about these putative differences. OLFM-4 knockout mice have a protective phenotype against sepsis [120], but the underlying mechanism(s) remain to be elucidated.

#### -CD177-expressing neutrophils

A similar dichotomy is present for CD177-expressing neutrophils [121]. CD177^bright^ cells are associated with immune regulation and play a potentially important role in antitumor immunity. These cells will also be discussed later.

#### -N1/N2 neutrophils

Neutrophils are found inside and around solid tumors. These tumor-associated neutrophils (TANs) have at least two flavors: N1 and N2 [122]. These two extreme phenotypes clearly differ with respect to antitumor responses. The functional consequences are highly relevant for antitumor immunity and will be discussed in more detail later.

#### -CXCR4-expressing neutrophils

Recently, mobilized blood neutrophils from the bone marrow do not express CXCR4 at such levels that it can be visualized by flow cytometry (results not shown and [60]). Several lines of evidence support the hypothesis that aged cells start expressing CXCR4 [60, 68, 123]. This is relevant, as the CXCR4-expressing cells will end up in the bone marrow, as this tissue is rich in the ligand of CXCR4, CXCL12 (formerly called SDF1α) [124], and will be cleared by efferocytosis. This hypothesis is supported by findings obtained in studies performed with patients with WHIM syndrome (warts hypogammaglobulinemia infections myelokathexis [125]) who have a gain-of-function mutation in the CXCR4 gene, rendering neutrophils hyperactivated by normal concentrations of CXCL12. Under these conditions, neutrophils cannot leave the bone marrow (myelokathexis). Treatment with antagonists of CXCR4, such as plerixafor, is associated with the mobilization of neutrophils and neutrophil progenitors from the bone marrow [60, 126]. These findings imply a pivotal role for CXCR4 in bone marrow retention in neutrophils.

#### -Segmentation of the neutrophil nucleus

A more old-fashioned method to determine the relative cellular age of neutrophils is the complexity of the nucleus of these cells. It is generally thought that the older the cells are, the more complex the nucleus becomes [127]. Indeed, combining stable isotope labeling with analysis of the nucleus led to the conclusion that cells with a banded nucleus are 2 days younger than neutrophils with a segmented nucleus [128]. Comparing neutrophils with normal segmented nuclei with cells with hypersegmented nuclei led to the surprising discovery that these latter cells were of similar ages. The function of the latter cells is immunoregulatory rather than pathogen killing [109]. The difference in function is also associated with a clear difference in the proteome, suggesting that both cell types are truly different subtypes resulting from parallel differentiation paths [128]. The existence of the parallel differentiation hypothesis is strengthened by the finding that CD16^bright^/CD62L^dim^ neutrophils are also found in the bone marrow (unpublished results). Unfortunately, this hypothesis awaits experimental support. If this suppressive neutrophil is a subtype that develops early in differentiation, it might be easier to target with therapy than if it were to be a more plastic phenotype occurring late after differentiation.

## Neutrophils in nonmalignant diseases

### Primary immunodeficiencies

The phagocytic compartment is evolutionarily old, which leads to a neutrophil compartment that is very robust [15]: very few primary immune deficiencies are based on mutations in the neutrophil compartment, and those that have been described are either rare and have severe functional and clinical consequences or are very mild and do not require clinical intervention. Mutations leading to neutrophil deficiencies are found in proteins associated with cytotoxicity [129–131] and differentiation/mobilization [132–134]. Currently, these diseases can be corrected by bone marrow transplantation [135] or even by gene correction [136]. Studying the effects of these mutations provides insight into the function of the neutrophil compartment and hence the role of neutrophils in tumor immunity.

### Neutrophils in inflammatory diseases

#### Disease caused by hyperactivation of the neutrophil compartment

In marked contrast to primary immune deficiencies, malfunction of the neutrophil compartment occurs in a multitude of proinflammatory conditions. Hyperactivation of neutrophils in vivo can lead to tissue damage, a detrimental effect that is typically found in proinflammatory diseases [137, 138]. This tissue damage is caused by the same toxic mechanisms that the cells utilize to kill pathogens [139]. Therefore, the immune system has a clear dilemma, as it should protect the host from invading pathogens via strong antimicrobial responses, while at the same time, the tissue should be protected from collateral damage. The preferred intended outcome is limited local inflammation, but occasionally, the situation cannot be eliminated, resulting in full-blown systemic inflammation that can be life-threatening [140]. Complete inhibition of the neutrophil compartment as a treatment option would be dangerous, as these cells are recruited with the important task of containing a pathogen threat. Thus, treatment for hyperactivation of neutrophils should focus on restoring homeostasis (balance) [2]. A similar concept also holds true in targeting neutrophils in cancer therapy. Excessive inhibition of the neutrophil compartment leads to compromised immunity against pathogens, increasing the risk for opportunistic bacterial infections [1]. An important extra complication is the likelihood that different neutrophil phenotypes are differentially sensitive to neutrophil-modulating therapy [141, 142] again, this issue will be discussed in more detail below, as it is important in the modulation of neutrophil responses in anticancer immunity.

#### Diseases associated with hypoactivation of the neutrophil compartment

In addition to diseases associated with hyperactivation of neutrophils, hypoactivation of the neutrophil compartment is a very dangerous condition, as it can jeopardize normal antipathogen responses. This condition is found in several severe clinical situations, such as traumatic injury, major operations and sepsis resolution. In this so-called compensatory anti-inflammatory response syndrome (CARS), patients become very sensitive to infectious complications that are associated with increased morbidity and mortality [141, 143]. Counterintuitively, CARS occurs in parallel with some characteristics of hyperinflammation, such as systemic inflammatory response syndrome or SIRS [142]. The simultaneous occurrence of SIRS and CARS leads to a ‘confused’ immune response, sometimes referred to as mixed antagonist response syndrome or MARS [144]. Clearly, MARS treatment should be based on both the activation of suppressed cells and the inhibition of activated cells. Little is known about whether different neutrophil phenotypes are differentially involved in SIRS and CARS. The example of MARS again illustrates that therapy should focus on regaining a balanced immune system rather than merely inhibition or activation.

Not only can clinical conditions lead to immune suppression, but several therapeutic interventions can also lead to hypoactivation of the neutrophil compartment. This is particularly evident in the use of cytotoxic drugs for the treatment of malignancies and hyperinflammation (when anti-inflammatory therapy does not suffice). A minimal threshold value of 0.5 × 10^9^ neutrophils/l of blood is generally applied to maintain sufficient protection against pathogens [145]. Interestingly, this cutoff value is based on clinical data but not on a biological mechanism. This cryptic statement relates to the fact that similarly low neutrophil counts can be found in the peripheral blood of people of African descent with a mutation in the duffy antigen. This condition is now termed duffy-null associated low neutrophil counts, as these people are completely healthy [146]. There seems to be no general correlation between blood neutrophil counts and the ‘fullness’ of the neutrophil compartment in the body (outside the peripheral blood) [9]. This finding is important for understanding the role of neutrophils in cancer immunity.

### Targeting the neutrophil compartment in disease

In conclusion, earlier sections clearly indicate that neutrophils are a ‘double-edged sword’ type of immune system that makes the neutrophil compartment complex. Traditionally, immune-mediated diseases are treated with inhibitors (‘antagonists’), as the use of activators (‘agonists’) is thought to be too dangerous because of the risk of collateral damage. Directly and specifically inhibiting neutrophil functions is very difficult. To date, no neutrophil-specific inhibitors are available for application in clinical practice. Importantly, broad anti-inflammatory therapies, such as those based on glucocorticosteroids, have only limited effects on neutrophil-driven inflammatory responses [147]. In fact, previous studies have suggested that gluco-corticosteroids promote the survival of neutrophils rather than their apoptosis [148].

Currently, anti-neutrophil therapy is based mainly on *in*direct inhibition of neutrophil-activating pathways by different biologicals. Good examples include anti-IL-8 (CXCL8) and blocking antibodies against cytokines that belong to the IL-17 pathway. Through these therapies, the redistribution and activation of neutrophils are affected. Unfortunately, in inflammatory diseases, no biologicals that directly target neutrophils have found their way to the clinic. The situation in cancer treatment seems different and will be discussed below. The main direct anti-neutrophil therapy clinically available is the use of cytostatic drugs, such as azathioprine and cyclophosphamide, which, at lower concentrations, can be used to at least in part directly inhibit neutrophils [149].

In contrast to these cytostatic drugs, two molecules are used to mobilize neutrophils from the bone marrow: granulocyte‒colony stimulation factor (G-CSF) [150] and the CXCR-4 antagonist plerixafor [151]. Very little, if any, solid clinical data are available for the use of these mediators in patients with CARS/MARS-type suppressed neutrophil conditions.

## Neutrophils as protumorigenic effector cells in tumors

### Neutrophils in and around the tumor

It has long been known that neutrophils are present in and around several types of tumors. These neutrophils are generally referred to as tumor-associated neutrophils or TANs [152]. It has also been well described that the presence of large numbers of TANs is associated with both positive and negative disease outcomes, depending on the type of tumor [153]. It was initially very unclear whether neutrophils could directly promote tumor progression or whether neutrophils merely reflect a response to the increased tissue damage caused by severe disease. The tissue damage found in tumors is associated with the release of damage-associated molecular patterns (DAMPs) [154, 155]. These DAMPs are very potent activators of innate immune cells, including neutrophils [156, 157]. Given that neutrophils are crucial in protecting against pathogens, anti-neutrophil therapy should be considered only if neutrophils have a direct protumorigenic effect. Numerous mechanistic studies in mice have confirmed such direct protumorigenic effects of neutrophils; however, the association between neutrophils and negative disease outcomes in humans likely reflects both explanations in parallel. In recent years, new evidence has been published that better describes the role of neutrophils as protumorigenic immune cells [158].

### Neutrophilic myeloid-derived suppressor cells (N-MDSCs)

Gabrilovich and colleagues proposed that early myeloid cell differentiation can occur around solid tumors and is associated with the suppression of local antitumor immunity [159]. Initially, MDSCs were defined on the basis of their propensity to suppress T-cell proliferation and activation [113, 159, 160]. This suppression was subsequently recognized to be mediated by at least two separate types of MDSCs, one of monocytic origin (monocytic MDSC) and the other of neutrophil origin (neutrophilic (or granulocytic) N-MDSC). Several studies have provided evidence for two main mechanisms underlying the inhibition of T cells by N-MDSCs: the depletion of arginine by the release of arginase-1 [161, 162] in the T-cell environment and the production of ROS in the immune synapse between MDSCs and T cells by a membrane-bound NADPH‒oxidase complex (NOX2) [111, 118]. The latter interaction requires the formation of an immune synapse facilitating close contact between N-MDSCs and T cellcells to allow short living ROS to interact with T cells [111]. N-MDSCs have been particularly studied in murine tumor models and relatively poorly studied in human studies, with the exception of patients with head and neck cancer. Brandau and colleagues provided good evidence that these cells are found in large numbers in these patients and that their presence is of clinical importance [161, 163]. Importantly, they showed that marked differences between the data of different laboratories are caused by differences in the technical workflow. More recent studies have suggested that these cells might be identified by new markers, including CD11b^+^ CD15^+^HLA-DR^low^CD66b^+^ [164]. Mouse N-MDSCs are characterized by CD11b^+^ Ly6G^+^ Ly6C^low^ [164]. Unfortunately, these combinations of markers on both mouse and human N-MDSCs are also found in conventional “mature” neutrophil phenotypes found in healthy individuals. More recently, LOX-1 and S100A9 have been proposed to define MDSCs. A recent study identified novel markers of N-MDSCs [165]. In-depth analysis led to the discovery of new markers of N-MDSCs found both in the bone marrow (mobilized by G-CSF) and in patients with tumors. Detailed bulk RNA sequencing led to the description of N-MDSCs as cells that express CD52, CD84 and the prostaglandin E_2_ receptor (PTGER-2) [165]. Thus, a consensus N-MDSC signature is lacking [164].

This lack of well-defined signatures of N-MDSCs leads to difficulties in the comparison and interpretation of many studies where only markers were used and the suppressive capacity was not simultaneously tested to show the involvement of MDSCs in immune responses. The reverse is also true: studies define MDSC-like activity without associations with clear markers. This makes comparisons between suppressive cells in different studies very difficult. It is unclear how much overlap exists between the different neutrophil subsets that can suppress T cells. Consequently, there is no agreement on how many types of N-MDSCs actually exist and their state of (im)maturity [113]. This lack of well-defined expression signatures has become even more relevant since, in recent years, other neutrophil phenotypes that inhibit T-cell responses have been identified [106, 111]. These cells have MDSC-like features, with the marked difference that they also have characteristics of normal mature cells, such as CD16^high^, CD10^high^ and CD62^low^ [106, 111]. Studies on MDSCs also suggested that at least some N-MDSCs have a mature phenotype [118].

Until a consensus is reached that a certain set of markers truly distinguishes a functional suppressive neutrophil phenotype, comparisons between different MDSC studies should be performed with caution, as differences in outcome might not be based on differences in pathogenesis but on technical issues. A future aim should be to try and reach a consensus on whether these are the same neutrophils in different settings and to agree on markers to use for identification.

Studies of N-MDSCs are particularly common in the cancer field. However, immune-suppressive neutrophils are also found in humans under inflammatory conditions. Here, the cells are named differently, and it remains unclear whether these cells (as discussed below) are different or actually the same [166]. To make collaborative progress, studies that investigate immune-suppressive neutrophils should at least adhere to two criteria: demonstrable suppression of T cells *and* characterization by certain markers. Several studies have met these two criteria:

#### Suppressive neutrophils are characterized by the CD11b^bright^/CD11c^bright^/CD62L^dim^

Antitumor responses are characterized by immune mechanisms typical for chronic conditions and seem to be modulated mainly by N-MDSCs. However, it is possible that suppressive neutrophils characterized by CD11b^bright^/CD11c^bright^/CD62L^dim^ that are quickly mobilized during acute and chronic inflammation [111, 167] are also involved in the suppression of T-cell responses in and around tumors [168]. Although this concept has not been directly tested, CD62L^low^ neutrophils have been connected with immune suppression in and near tumors [169]. L-selectin is a complex marker because it is quickly shed upon stimulation of cells [170]. This makes it difficult to discriminate between CD62L^low^ cells originating from the bone marrow and cells that have been activated near or in the tumor. However, CD62L^low^ cells have been shown to be potent suppressors of T-cell function through the formation of an immune synapse and the production of ROS [111, 171]. This finding is very reminiscent of the suppressive mechanisms described for N-MDSCs. Notably, CD11b^bright^/CD11c^bright^/CD62L^dim^ cells are not immature but are similar in age to normal neutrophils and are 2 days older than banded neutrophils [128].

#### Suppressive neutrophils characterized by differential expression of CD10

CD10 (also called neprilysin, CALLA, neutral endopeptidase and enkephalinase) is a complex marker, as it is both a marker of late differentiation [107, 172] and an activation marker [108, 173]. The enhanced expression of CD10 upon stimulation in vitro follows the expression of Mac-1 (CD11b/CD18), suggesting that both markers are expressed in the same subcellular compartment [108, 174]. In addition to being expressed on the cell surface at low levels in resting neutrophils, intracellular Mac-1 is located in secretory vesicles and specific granules [175]. Upon activation, both markers are quickly expressed at high levels on neutrophils; therefore, Mac-1 is often used as a general activation marker for neutrophil activation both in vitro and in vivo.

Upon treatment of healthy donors with G-CSF, massive amounts of CD10^dim^ (‘young’ cells) and CD10^bright^ (mature cells) cells are mobilized from the bone marrow [106]. The two populations have opposite effects on T-cell proliferation, where CD10^bright^ cells have suppressive characteristics, whereas CD10^dim^ cells have activating properties. Recently, CD66b^+^CD10^+^CD16^+^CD11b^+^ immunosuppressive neutrophils/N-MDSCs have been better phenotyped because N-MDSCs are characterized by differential expression of CD52, CD84 and PTGER2 [165]. These cells are implicated in antitumor responses, as the phenotype has been found in tumor-associated neutrophils (TANs) [165]. The general idea put forward by the authors is that these TANs undergo specific reprogramming once they reach the tumor site. It is still an open question whether these cells are protumorigenic and should be targeted or whether the cells are bystander cells that respond to DAMPs released from the tumor. Interestingly, in the same study, CD16^high^/CD62L^dim^ cells isolated from the blood during acute systemic inflammation did not exhibit this N-MDSC profile [165].

#### CD177(NB1)^±^ neutrophils

The neutrophil compartment in healthy individuals is characterized by bimodal expression of the GPI-linked protein CD177 [121, 176]. The importance of CD177 is unclear, as individuals deficient in this protein (3–10% of the general population) are healthy [177]. CD177^bright^ neutrophils have been implicated in the host immune response to different solid tumors [178–180]. However, mixed results have been reported in terms of disease outcome. In colitis-associated cancer and colorectal cancer, the presence of CD177^bright^ neutrophils is associated with better overall and disease-free survival [178], whereas in pancreatic duct adenocarcinoma, increased numbers of CD177^+^ neutrophils in the tumor are associated with a poor prognosis [180]. These diverse findings might indicate a complex ‘cause and consequence’ situation. As indicated in the introduction, DAMPs produced by highly malignant tumors might attract CD177^+^ neutrophils as a consequence of tissue damage, which does not necessarily place these neutrophils in the pathogenesis of these tumors.

#### N1/N2 tumor-associated neutrophils

Fridlender and colleagues described a dichotomy in TANs found in and around solid tumors [152, 181]. They provided evidence in mice that neutrophils arriving near the tumor side can develop into antitumorigenic N1 cells or protumorigenic N2 cells. The choice between the two seems to be dependent on the production of transforming growth factor (TGF)β in the tumor environment, which promotes protumorigenic N2 cells [122, 170]. This differentiation is induced by local tissue/tumor cues and can be considered plasticity rather than a bona fide subtype.

#### PD-L1-expressing neutrophils

Interestingly, both in humans during acute inflammation [182] and in cancer patients [81], a neutrophil phenotype with relatively high expression of PD-L1 was observed. In the acute inflammation model, this subset presented an IFN-induced transcription profile. Indeed, high IFN-γ concentrations could induce the expression of PD-L1 on neutrophils from healthy controls in vitro [182], by which the cells were additionally shown to inhibit the cytotoxicity of T cells.

#### Multiple phenotypes based on single-cell RNA-seq

Single-cell RNA sequencing is a great tool for studying heterogeneity within a pool of cells. However, because neutrophils have relatively low levels of RNA and high levels of RNase, single-cell RNA sequencing is challenging for neutrophils. Recently, much progress has been made in this area; therefore, this field is rapidly expanding. Single-cell RNA sequencing revealed multiple phenotypes of TANs in human non-small lung cancer [183] and pancreatic cancer [184]. Xue et al. provided evidence that differences in the liver tumor microenvironment steer the development of at least 6 different TANs in humans [81]. In an elegant study by Wu et al., human neutrophils were analyzed across 17 different cancers, and ten different neutrophil states were detected, among which the states of inflammation, angiogenesis and antigen presentation were dominant across different cancers [185]. By clustering analysis, one can infer ‘pseudotime’, which is not a representation of time per se but rather how likely it is that one subset differentiated from another subset. Two studies proposed a continuous differentiation trajectory along neutrophil states [81, 185], whereas other studies reported less linear plasticity [183, 184]. In one study, antigen-presenting neutrophils were terminally differentiated [185], whereas in another study, this subset was still plastic [183]. Importantly, nontumor-associated neutrophils were found to differentiate into tumor-associated neutrophils [183, 184], supporting the concept that the tumor microenvironment can induce plasticity.

These data clearly illustrate the enormous plasticity of neutrophils in general and TANs in particular. Single-cell RNA-seq and pseudotime analysis hold great promise for better predicting disease outcomes and for therapeutically targeting specific differentiation pathways. However, because neutrophils from different clusters cannot yet be sorted, it is impossible to validate their function in functional assays at this time. To be able to isolate these cells would be an important step forward in the field.

## Neutrophils promote tumor growth

**Fig. 1:**
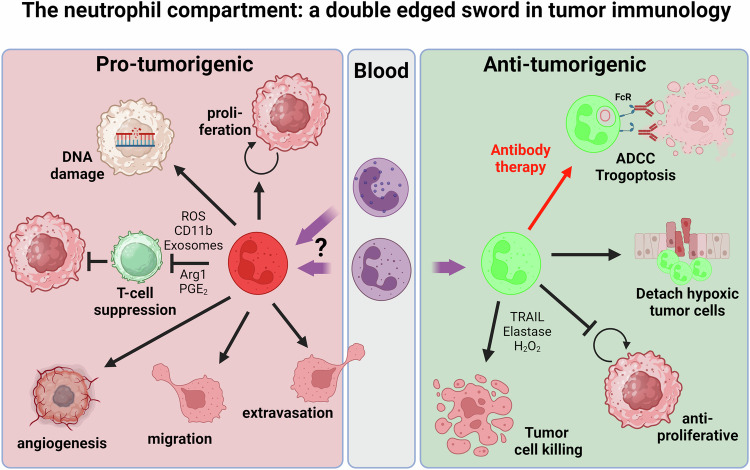
Neutrophils and tumor immunology : a double-edged sword. Neutrophils are involved both in antitumor defense and protumorigenic effector cells. Most data in the literature support the hypothesis that homeostatic neutrophils are involved in tumor killing mediated by mechanisms normally used in antipathogen defense. The protumorigenic responses are more based on cells that transdifferentiate into immune-suppressive cells in response to cues originating from the tumor microenvironment. Additionally, neutrophils have direct effects on different stages of tumor progression. Made with biorender

In addition to the suppression of the antitumor immune responses mentioned above, neutrophils are directly involved in promoting tumor growth via additional mechanisms (Fig. 1**)**.

### Neutrophils promote tumor cell proliferation

Evidence for direct effects of neutrophils on tumor proliferation mainly comes from animal models but is supported by associations in human cancer patients. In zebrafish, neutrophils interact with single preneoplastic cells and, by subsequently releasing the trophic factor prostaglandin E_2,_ support preneoplastic cell outgrowth [186, 187]. In line with these findings, another study in zebrafish showed that amplifying the innate immune response via the induction of a wound near the tumor was associated with increased proliferation of preneoplastic cells. The same authors reported a correlation between neutrophil influx and the proliferative index of tumor cells in human melanomas with superimposed ulceration wounds [188]. Therefore, it is tempting to speculate that the poor survival of melanoma patients with high numbers of tumor-infiltrating neutrophils [189] is in part due to this neutrophil-induced proliferation of tumor cells.

Another protein associated with human tumor progression is neutrophil elastase [190]. In mouse models of lung and breast cancer, the deletion of neutrophil elastase led to a reduction in tumor cell proliferation, implying a direct effect [191, 192].

### Neutrophils promote DNA damage

Defects in the DNA repair mechanisms of cells can cause genetic instability and mutations, which can cause tumor cells to grow and metastasize. For example, the number of double-stranded DNA breaks has been found to increase in human intramucosal neoplasia and invasive carcinoma [193]. In mice, neutrophils have also been shown to directly influence tumors by promoting double-strand DNA breaks in intestinal epithelial cells. Neutrophil-derived microparticles were found to contain two microRNAs, miR-23a and miR-155, which inhibit the production of a critical DNA repair protein. Moreover, the severity of colonic inflammation is an important determinant of the risk of colorectal neoplasia in human patients with ulcerative colitis [194], suggesting a role for neutrophils.

### Neutrophils promote angiogenesis

As discussed above, angiogenesis is an important mechanism during wound healing that can be hijacked by tumors. When a tumor grows beyond a certain size, angiogenesis is pivotal for providing oxygen and nutrients to sustain tumor growth. Angiogenesis also provides a means for tumor cells to enter the blood circulation and metastasize at other tissue locations [195]. Many studies have implicated a role for neutrophils in angiogenesis. In hepatocellular carcinoma patients, infiltration of peritumoral neutrophils is positively correlated with angiogenesis progression at the tumor-invading edge and predicts poor survival [196]. Mechanistically, matrix metalloproteinase 9 (MMP-9) from neutrophils can stimulate angiogenesis in vitro [196], and using MMP-9 knockout mice, a direct relationship was found between neutrophil MMP-9 and increased tumor angiogenesis in a colorectal cancer model. In this model, MMP-9 acts by degrading the extracellular matrix to release vascular endothelial growth factor (VEGF), thereby inducing angiogenesis [197]. In addition, neutrophils were also found to promote tumor angiogenesis in mouse models of pancreatic cancer [198, 199]. Interestingly, using single-cell RNAseq, Ng et al. reported a distinct neutrophil state with a proangiogenic function in a mouse model of pancreatic cancer. This distinct neutrophil stage can also be found across multiple tumor types in humans, and importantly, human cancer patients with high expression of this neutrophil state have poorer overall survival [184]. Similar results were reported in another recent single-cell RNAseq study, which revealed that the angiogenic neutrophil state was one of the most dominant states across different cancer types and was correlated with a worse disease outcome [185]. Taken together, these in vitro and in vivo data suggest a causal relationship between neutrophils and angiogenesis in tumors. The associated poorer survival could be due to a larger primary tumor, an increase in metastasis or both [196].

## Neutrophils promote metastasis

**Fig. 2:**
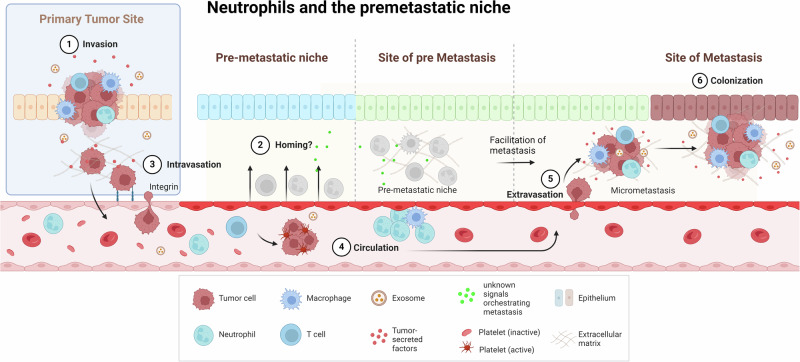
Neutrophils and the premetastatic niche: an issue of cause and consequence. Neutrophils are involved in the guidance of metastatic cells originating from the tumor into a preferred premetastatic niche in distant tissue. Here, two nonmutually exclusive mechanisms mediated by neutrophils drive metastasis: i. Homing of tumor cells is an intrinsic response: neutrophils facilitate the growth of the metastasis or ii. Neutrophils control the preparation of tissue to facilitate the homing of metastatic cells to the tissue. The end result of these two hypotheses is that TANs facilitate metastatic disease. Made with biorender

For a tumor cell to metastasize, it needs to detach from the primary tumor, start migrating, intravasate into the blood vessel, extravasate at a distant site and start growing (Fig. 2). Neutrophils play a role in promoting tumor cell detachment and migration, the formation of the premetastatic niche and the awakening of dormant cancer cells at distant sites. This fascinating research area studies the underlying processes that can explain the preference of metastatic cells for certain tissue locations. Understanding this process is critical, as long-term cancer therapy should focus on controlling micrometastases and dormant cancer cells “waiting” to proliferate in the future.

### Tumor cell motility

Tumor cell motility is one of the hallmarks of tumor progression, as it facilitates both tumor invasiveness and metastasis. Epithelial tumors are firmly held together and first undergo epithelial-to-mesenchymal transition to detach and migrate away from the primary tumor [200]. Neutrophils play a role in this first phase of detachment [201, 202]. In another in vitro study in humans, both IL-17 production and neutrophil elastase production by neutrophils were implicated in the epithelial-to-mesenchymal transition of tumors [203]. Moreover, human neutrophils were shown to promote tumor cell migration in a human hepatocellular carcinoma cell line in vitro [204]. In addition to epithelial tumor cells, the migration of primary human glioblastoma cell lines was increased by human neutrophils in vitro. Direct contact was not necessary for this increase in migration, suggesting that factors secreted by neutrophils induce this migratory effect. Using repetitive intravital imaging of mouse gliomas in the brain, it was found that a biopsy wound increased glioma tumor cell migration, which could be prevented by depleting neutrophils [205]. Together, these studies suggest a direct role for neutrophils in the detachment of the primary tumor and in tumor cell motility.

### Extravasation

Next, tumor cells need to infiltrate the blood, extravasate from the circulation and enter another tissue at a distant site. In the blood circulation, tumor cells form a triad with circulating neutrophils and endothelial cells. A flow migration assay demonstrated that LFA-1 on human neutrophils initially captures ICAM-1 on both melanoma cells and the endothelium, whereas neutrophil Mac-1 binding to ICAM-1 stabilizes the aggregation [206]. Another study confirmed that neutrophils mediate increased extravasation of a human melanoma cell line in an in vitro microfluidics system as well as in zebrafish [207]. Neutrophil clustering with circulating tumor cells has also been detected in human breast cancer patients, and patients harboring such clusters have a lower survival rate [208]. Another way by which neutrophils facilitate extravasation of tumor cells is the production and release of IL-1β, which activates the endothelium and results in concomitant MMP9 production, as shown in a mouse model of melanoma metastasis [209]. Thus, evidence suggests that neutrophils can aid in the extravasation of tumor cells in in vitro and animal models in vivo. Additional evidence in humans suggests that this mechanism also plays a role in human metastasis.

### Premetastatic niche

As early as 1889, Steven Paget introduced the “seed and soil hypothesis”, in which cancer cells were hypothesized to alter the microenvironment [210]. David Lyden et al. extrapolated this concept by suggesting that primary tumors can influence the microenvironment at distant organ sites to help initiate and promote metastasis [211]. Figure 2 visualizes the current understanding of the concept. Currently, researchers agree that the premetastatic niche can promote tumor growth; however, whether the premetastatic niche is an absolute requirement for metastasis formation is still under debate.

Neutrophils are elevated in the circulation of humans with progressive cancer [212, 213], and a significant proportion of the cells in the premetastatic niche in both mice and humans [214, 215]. Similarly, G-CSF-induced mobilization of neutrophils from the bone marrow was shown to be essential for the formation of the premetastatic niche in mice. G-CSF-mobilized neutrophils subsequently secrete Bv8, which is a protein involved in both angiogenesis and further recruitment of neutrophils to the metastatic site. Both anti-GCSF and anti-Bv8 agents significantly reduce lung metastasis [216], suggesting a direct role for neutrophils. In addition to the protein Bv8, neutrophils produce S100A8 and S100A9 in the premetastatic niche [217–219]. Interestingly, these molecules can not only further increase neutrophil recruitment [216] but also promote metastasis formation [217]. As for Bv8, targeting S100A8 and S100A9 with neutralizing antibodies reduces metastasis formation [220]. In vitro experiments revealed that Bv8, S100A8 and S100A9 could all stimulate tumor cell migration [217], indicating a direct role for these molecules in both neutrophil and tumor cell migration. Alternatively, neutrophil-derived MMP9, as well as S100A8 and S100A9, influences tumor cell survival at the metastasizing site [220]. In line, high S100A8 and S100A9 expression in the metastatic nodules of human breast cancer patients predicts shorter survival [219].

Another molecule secreted by neutrophils that is involved in the premetastatic niche is leukotriene B_4_. Neutrophil-derived leukotriene B_4_ binds leukotriene receptors on mouse breast tumor cells to expand a subpopulation of cancer cells that are good initiators of metastasis. In the same study, leukotriene receptor expression was also found in human breast carcinomas and their lymph node metastases [221], implying a similar role for leukotriene B_4_ in humans.

The CXCR4‒CXCL12 axis, which in homeostasis is connected to bone marrow retention and the clearance of aged CXCR4-expressing neutrophils in the circulation, is also involved in the premetastatic niche. The premetastatic niche in the liver was found to contain increased levels of CXCL12, which recruits neutrophils via a CXCR4-dependent mechanism [222]. The inhibition of CXCL12/SDF-1, as well as neutrophil depletion, reduces liver metastasis, indicating a direct role for neutrophils [222]. The increased CXCL12 could be linked to increased tissue inhibitor of metalloproteinases (TIMP)-1 levels, and TIMP-1 levels in plasma correlate with liver metastasis in human colorectal cancer [222].

Another study reported that cathepsin C secreted by primary tumors facilitates interleukin-1β (IL-1β) processing in neutrophils, which in turn results in NETosis in the lung. Proteases in this type of extracellular DNA can degrade the metastasis-suppressing extracellular matrix protein thrombospondin-1. In line, cathepsin C secretion is associated with extracellular DNA and lung metastasis in human breast tumors [223].

In all the aforementioned studies, it has not been clarified whether neutrophils reside in the vasculature of the premetastatic organ or actually extravasate and enter the tissue. Neutrophils have been shown to make the lung vasculature leaky, resulting in increased metastasis [224], which might also aid in the extravasation of both neutrophils and tumor cells. However, formal evidence of extravascular neutrophils arriving before metastasizing tumor cells is lacking (Fig. 2).

The increase in neutrophils in the circulation might facilitate the formation of the premetastatic niche. For years, clinical studies have consistently shown that the neutrophil/lymphocyte ratio in the peripheral blood is an interesting diagnostic tool associated with the severity of oncological disease [212, 213]. However, this could also simply reflect that more malignant or high-volume tumors will produce more DAMPs and more systemic inflammation. Systemic inflammation is associated with increased neutrophil numbers and decreased lymphocyte numbers. In fact, this systemic inflammation might be involved in the creation of a premetastatic niche.

### Awakening of dormant metastatic tumor cells

Metastasized tumor cells can remain dormant at distant sites, where they remain viable but fail to proliferate. These dormant cells can be triggered to proliferate at a later time point to form actively growing metastases. Albrengues et al. [225] reported that lung inflammation-induced neutrophil extracellular traps (NETs) could trigger dormant metastatic cells in mice. Neutrophil elastase and MMP9 in the extracellular DNA sequentially cleave the extracellular protein laminin, revealing an epitope that triggers the proliferation of dormant metastatic tumor cells in the lungs of mice. Inhibiting or digesting NETs or an antibody against the exposed epitope of laminin indeed prevents dormant tumor cells from growing [225]. To date, evidence for this principle in humans has not been reported.

## Natural antitumor response of neutrophils

In addition to their many protumorigenic properties mentioned above, neutrophils also exhibit natural antitumor responses. However, the number of studies reporting the natural antitumor effects of neutrophils in vivo is limited:

### Antiproliferative effects of neutrophils

As early as 1988, a cytostatic effect of neutrophils on tumor cells was reported. After being treated with rTNF, human neutrophils were found to induce the cytostasis of numerous human tumor cell lines in vitro. A hydrogen peroxide scavenger, but not other ROS inhibitors, could counteract this effect [226]. Another study revealed that direct cell contact between Fas ligand (FasL) on human neutrophils and Fas on tumor cells could inhibit the cell cycle of tumor cells in vitro [227].

### Debridement of hypoxic tumor cells

Interestingly, Blaisdell et al. [228] reported a surprising mechanism by which neutrophils can halt tumor growth and metastasis in a mouse model of PTEN-deficient uterine epithelial cancer. Hypoxic tumor cells induce neutrophil infiltration and subsequent detachment of the basement membrane of these tumor cells. Similarly, PTEN-deficient uterine cancer is a cancer type in which neutrophils are correlated with improved survival in humans [228].

### Apoptosis/direct tumor cell killing

Several effector molecules have been implicated in the killing of tumor cells by neutrophils. For example, tumor necrosis factor-related apoptosis-inducing ligand (TRAIL) selectively induces apoptosis in tumor cells, and the increased expression of TRAIL by neutrophils has been shown to induce the apoptosis of leukemic T cells in vitro [229]. The selective killing of tumor cells by human neutrophils in vitro and in vivo in mice also occurs via neutrophil elastase, which proteolytically releases the CD95 death domain [230].

In another study, only neutrophils from mice bearing tumors, but not neutrophils from healthy mice, were found to kill tumor cells in H_2_O_2_-dependent matter requiring direct cell contact. Similarly, neutrophils from human breast cancer patients also showed increased killing of tumor cells compared with neutrophils from healthy volunteers [231].

## Harnessing neutrophils for antitumor therapy

Although many studies have reported that depleting neutrophils in mice can rigorously decrease metastasis formation [232–234], inducing neutropenia in humans as a therapeutic option is life-threatening and not sustainable. Here, we discuss other options to harness or target neutrophils for antitumor responses.

### Inducing ADCC and trogocytosis by treatment with antibodies against cancer

When a tumor cell is decorated with antibodies, the binding of these antibodies to Fc receptors on adjacent neutrophils can trigger antibody-dependent cell-mediated cytotoxicity (ADCC). After this FcR recognition of antibodies on the target tumor cell, the neutrophils start nibbling pieces of the membrane of the tumor cell (trogocytosis), leading to a type of cell death termed trogoptosis [235, 236]. Thus, finding the right target antibody against cancer cells could induce ADCC in neutrophils and be used as a treatment.

Neutrophils express both Fcα and Fcγ receptors, which can bind to the Fc portion of IgA and IgG antibodies, respectively [11]. The low- to intermediate-affinity IgG Fc receptors FcγRIIIB (CD16) and FcγRIIA (CD32) are constitutively expressed on neutrophils, whereas the high-affinity Fc receptor FcγRI (CD64) is only upregulated in the presence of IFN-γ or G-CSF [237, 238]. The Fc receptor that binds to IgA is FcαRI (CD89) [12], which is also constitutively expressed on neutrophils [12]. The functionality of these receptors is well regulated by several inflammatory mediator-induced signals [239]. Therefore, optimal activation of FcγRIIA [240], FcγRI [241] and FcαR [242] by their ligands is sensitive to inside-out signals induced by cytokines, chemokines and other inflammatory mediators. This finding implies that the optimal function of FcRs is dependent on the presence of proinflammatory cytokines in the vicinity of the cells that express these receptors [239]. This inside-out control is also found in the function of integrin receptors, such as those expressed on neutrophils (e.g., Mac-1 CD11b/CD18) [243]. There is clear synergism between FcRs and integrins in the function of neutrophils in response to serum opsonized targets [244]. Furthermore, coactivation of complement with immunoglobulins can facilitate tumor killing, although it might cause proinflammatory complications necessitating early termination of treatment [245]. It is tempting to speculate that fine-tuning the internal control of complement and immunoglobulin receptors might lead to optimal killing of tumor cells with minimal collateral damage caused by inflammation [239, 243].

Many studies have reported that antibodies can induce ADCC in human neutrophils in vitro [246]. It is indeed not surprising that decorating a tumor cell with antibodies induces ADCC by neutrophils when they are placed in the same culture dish. The in vivo situation, however, is much more complex. The ability of the antibodies to penetrate the tumor tissue and bind the tumor cells, as well as the number and location of neutrophils and other effector cells within the tumor, greatly influences the amount of neutrophil-mediated ADCC. The translatability and practicality of in vivo studies in mice are complicated by the fact that murine neutrophils bear different FcγRs [10] and lack FcαRs [11, 12]. Nevertheless, a specific role of neutrophil ADCC has been reported after the administration of therapeutic antibodies in mice [247]. The first monoclonal antibody (mAb) approved to treat human cancer was rituximab in 1997. Rituximab is an anti-CD20 antibody that is used to successfully treat B-cell lymphoma [248]. The role of neutrophils in this antitumor effect in vivo remains to be established. Unfortunately, the treatment of other solid tumors with mAbs has proven to be more difficult. New strategies to improve mAb treatment are continuously explored.

These antibody-based therapies are under intense development, as multiple manipulations of antibodies can lead to greatly increased efficacy. Examples include bispecific antibodies for improved (co)-crosslinking [249], bispecific antibodies to combine IgA- and IgG-based killing [250] and mutations in the Fc-tail [251]. Most, if not all, therapeutic antibodies are based on immunoglobulin G binding to FγRs. However, recent work argues that therapeutic IgA antibodies might work better in neutrophil-mediated ADCC, as these antibodies better attract neutrophils toward the tumor via poorly defined mechanisms [252, 253].

An important issue in FcR-mediated killing of tumor cells in vitro and in vivo is the genetic background of the patient. Several polymorphisms in the FcR genes [254, 255] can play important roles in this process. An interesting example is FcγRIIa, with the higher affinity variant H131, which responds better to treatment with anti-HER2 antibodies than patients with the R131 variant of this gene [256, 257].

### A possible role for exploiting inside-out control

As indicated above, FcRs and complement receptors play important roles in antitumor therapy. These receptors are present on innate immune cells that have the armamentarium to kill pathogens and tumor cells but unfortunately kill healthy host tissue alike. Therefore, these cells are under tight control by inside-out signals that determine the activity of these receptors. These signals are crucial for the fine-tuning of their functionality: sufficient activation for killing pathogens with as little collateral damage to the host tissue as possible. It is only logical to find therapeutic ways to release this toxic power of neutrophils near or inside a tumor by liberating positive inside-out signals. These positive signals are generated by multiple mediators, such as DAMPs, bioactive lipids, chemokines and cytokines, that can be used to optimize or fine-tune therapies on the basis of the use of Fc-receptors.

Tumors and pathogens can be opsonized by both immunoglobulins and complement fragments; therefore, it is not surprising that both the Fc- and complement-receptor families interact with each other, resulting in a very flexible response [258–260]. Complement receptor 3 is an integrin that is also called Mac-1 and is also important in adhesion and transendothelial migration [252, 261]. It is plausible that antitumor therapy based on therapeutic monoclonal antibodies can be improved not only by engineering the antibodies but also by manipulation of the antitumor response by priming neutrophils such that both FcRs and complement receptors are in an optimal configuration to convey ADCC. An alternative approach to aid ADCC is therefore to target the machinery of inside-out control, such as by inducing the open configuration of integrins [262] or by manipulating intracellular signals such as FERMT3 [133] or kindlin-3 [263].

### Targeting inhibitory receptors

Interestingly, neutrophils also express multiple inhibitory receptors important for the local control of neutrophil effector responses, which might be exploited in antitumor cell therapy. The functions of these immune checkpoints, e.g., SIRL [264], LAIR [265], PD1/PDL1 [182], and SIRP1-alpha/CD47 [266], must be considered when designing treatments that utilize neutrophils to treat tumors, as these molecules can counteract tumor killing by neutrophils. Therefore, for proper antitumor responses by neutrophils, manipulation of these inhibitory receptors might be needed.

### Targeting the premetastatic niche

A fascinating scientific area is the putative targeting of the formation or presence of a premetastatic niche. The inhibition of the mechanisms by which tumor cells disseminate from the primary tumor and home to premetastatic niches holds great therapeutic promise, as metastatic disease is the main cause of mortality in cancer patients. Both G-CSF and Bv8 have been implicated in the premetastatic niche, and anti-GCSF and anti-Bv8 significantly reduce lung metastasis in mice [216]. In particular, Bv8 might be an interesting target, as its effects are likely more confined than those of G-CSF. Furthermore, S100A8 and S100A9 were shown to be involved in neutrophil and tumor cell motility as well as tumor cell survival [217], making them interesting targets for preventing metastasis. As the CXCR4/CXCL12 axis is also crucially involved in the bone marrow kinetics of neutrophils, this target is likely too drastic to be a viable option.

## Indirect roles of neutrophils in other cancer therapies

Chemotherapy to treat dividing cancer cells similarly causes decreased production of neutrophils in the bone marrow, resulting in extreme and life-threatening neutropenia [267]. On the other hand, successful immunotherapy with checkpoint inhibitors can induce immune-related adverse events characterized by increased neutrophil numbers in the peripheral blood. While these examples are just bystander effects, indirect positive and negative effects of neutrophils on other cancer therapies have also been reported.

### Indirect positive roles of neutrophils in other cancer therapies

Indirect roles of neutrophils have been reported in immunotherapies targeting other immune cells. Successful immunotherapy with anti-CD40 expanded a specific CD62L^hi^SiglecF^low^ neutrophil phenotype, resulting in an interferon gene signature in both mice and humans. In mice, the loss of the interferon-responsive transcription factor IRF1 in specific neutrophils was associated with failure of immunotherapy, demonstrating a crucial role for these cells in effective treatment. Similarly, an increase in neutrophils after checkpoint inhibitor immunotherapy in human lung cancer patients was positively correlated with improved therapeutic outcomes. However, the role of neutrophils with the interferon gene signature has not been investigated in these patients. Therefore, the increase in neutrophil counts after immunotherapy in human patients could also be a response to successful tumor damage. Regardless, increases in neutrophil counts upon therapy could be used as a predictor of the response to immunotherapy.

In a related study, interferon-stimulated Ly6E^hi^ neutrophils could predict the anti-PD1 response to mouse breast tumors and additionally directly sensitize otherwise unresponsive breast tumors to anti-PD1 immunotherapy after adoptive transfer in mice. Ly6E^hi^ neutrophils achieve this via the activation of cytotoxic CD8+ T cells through IL-12b secretion. In this study, they also examined Ly6E^hi^ neutrophils in human melanoma and lung cancer patients and reported that these cells displayed a gene signature similar to that of mice. Importantly, an increase in the number of Ly6E^hi^ neutrophils before treatment can accurately predict the immunotherapy response in humans. Furthermore, the predictive capacity of the Ly6E^hi^ neutrophil count outperformed that of the total neutrophil count, indicating that these neutrophils play a specific role in the response to immunotherapy in humans.

Another indirect role of neutrophils in immunotherapy is that of neutrophil antigen-presenting cells, which was introduced in the ‘Multiple phenotypes on the basis of single-cell RNA-seq’ section. A significant positive correlation was found between the HLA-DR+ antigen-presenting neutrophil signature and survival upon immune therapy in patients with different cancers.

### Mediators of therapy resistance

In addition to the positive roles of neutrophils in other cancer therapies mentioned above, neutrophils have also been demonstrated to negatively affect other treatments, for example, by inducing therapy resistance. The best studied example is the reduced effectiveness of anti-VEGF treatment due to the presence of neutrophils capable of boosting angiogenesis in mouse models [268, 269], although this is not yet recapitulated in humans. The ability of neutrophils to promote tumor resistance to radiation therapy has been reported both in a mouse model and in humans [270].

## Important dilemmas in targeting TANs in antitumor therapy

The role of human neutrophils in antitumor defense is difficult to address because the issues of ‘cause or consequence’ and ‘different influences of microenvironments in different tumors’ are very difficult to disentangle. Surely, more malignant tumors produce more DAMPs that attract more neutrophils. However, the fate of these cells in the tumor environment is complex, as they adapt to different tumor microenvironments depending on the inflammatory status of the patient, the type of neutrophil that is attracted, the microenvironment in and around the tumor, and the degree of tissue damage present in the necrotic tumor core. In fact, the question what the role of neutrophils is in antitumor responses is not specific enough and thus meaningless, as the cells behave differently depending on the type of neutrophil, the timing of the immune response and in response to the microenvironment.

### Plasticity of neutrophils

**Fig. 3:**
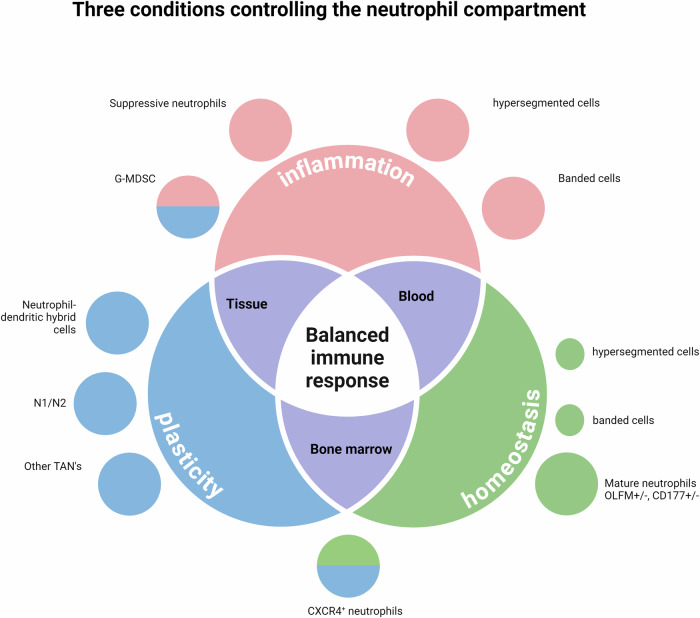
Control of the neutrophil compartment under different conditions from homeostasis in the blood to plasticity in the tumor microenvironment (TME). Green: Under healthy conditions, the vast majority of neutrophils are homeostatic cells that are directed against invading pathogens. In red, under conditions of self-limiting infection/inflammation, the neutrophil compartment responds with a bone-marrow response that is characterized by priming [281] and mobilization (neutrophilia) of neutrophil phenotypes involved in pathogen killing. When the inflammatory process is well controlled, such as during wound healing, tissue-dwelling neutrophils play a positive role in the tissue. In blue: When the tissue harbors uncontrolled and aberrant inflammation [282], such as that found in the TME, the differentiation and activation of neutrophils can lead to a protumorigenic microenvironment with suppressed antitumor responses. This chronic inflammatory response propagates chronic neutrophil responses, leading to a ‘confused’ immune response. Made with biorender

As also indicated in the section ‘Phenotypes’ and ‘Neutrophils as protumorigenic effector cells in tumors’, neutrophils in the tumor microenvironment cannot be separated according to the existence of clearly identifiable phenotypes. The situation is much better described as a spectrum of cells that adapt to heterogeneous signals from the tumor, such as those described in non-small cell lung cancer [183]. This heterogeneity precludes a therapy that only takes “protumorigenic neutrophils” into account. Targeting protumorigenic neutrophils while not compromising “antitumorigenic” neutrophils that coexist in the TME is a great therapeutic challenge. In addition, the treatment should not induce plasticity of antitumorigenic cells into protumorigenic cells (Fig. 3).

#### Induction of neutrophil–DC hybrids and neutrophil–APC (nAPC)

In the last decade, several studies have been published showing neutrophil plasticity via the induction of antigen-presenting cell characteristics, such as the expression of HLA-DR and antigen-presenting capacity [271–273]. Matsushima provided evidence that neutrophils can not only express HLA-DR but also cross-differentiate into dendritic cells and named these cells “neutrophil-DC hybrids” [271]. The potential importance of these cells was strengthened by in vivo experiments [272]. The concept of such hybrid cells was supported by the study of Mysore and colleagues, who showed how immune complexes and Fc-receptors drive conversion toward a dendritic type of cells [274]. These studies on neutrophil plasticity can have important implications, as such CD45^+^ cells present in the tumor and the TME can be misclassified as canonical dendritic cells. The result of such plasticity is difficult to predict, but it may instruct the T-cell compartment to start antitumor immunity. Targeting these cells ‘by mistake’ with anti-neutrophil therapy might have severe clinical consequences, particularly infectious complications.

#### Other phenotypes resulting from neutrophil plasticity

Dyugovskaya et al. [275] described giant neutrophils after 7 days of culture. Although these cells do not visually resemble mature neutrophils, they do express neutrophil markers and are devoid of monocyte markers. It is uncertain whether these cells play a role in the TME but is another example that the plasticity of neutrophils may lead to the occurrence of cells that might easily be missed in vivo, as they no longer exhibit typical neutrophil morphology.

Ogawa et al. [276] showed in the mouse in vivo plasticity of neutrophils in the olfactory neuroepithelium under conditions of inflammation and/or tissue injury. They elegantly showed that under these conditions, neutrophils can acquire eosinophil/basophil characteristics (SiglecF expression) while retaining the neutrophil marker Ly6G. In his commentary on this study, Ronen Sumagin rightly questions the consensus that neutrophils are by definition end-stage cells [277], as multiple studies show compelling evidence that neutrophils can change in response to diverse tissue cues.

### The challenge of adopting neutrophil plasticity as important in the TME

Neutrophil plasticity has two major problems. First, plasticity leads to changes such that the cells no longer express typical neutrophil characteristics [273, 274]. This might lead to underestimation of the role of neutrophils in immune responses in the TME. Second, the data on neutrophil plasticity support the hypothesis that neutrophils change in different directions depending on the tissue cues that the cells encounter [183].

These hypotheses might have major consequences for targeting neutrophils in the TME, as it is completely uncertain how many ‘plastic’ forms of neutrophils are found in different tumor locations at different times. Specific targeting of these forms while keeping homeostatic neutrophil responses intact will be a major challenge if at all possible. This might require a personalized analysis of the primary tumor environment, but this can be completely different at the location of the major problem with cancer: the growing metastasis at different tissue sites. It is only logical to assume that heterogeneous metastatic disease present at different tissue locations will change neutrophil plasticity in different directions compared with the original neutrophil state in the original tumor. The identification of a specific feature present only in neutrophils in the TME of every different tumor is highly unlikely. On the other hand, specific personalized treatment of diverse neutrophils in different (micro)metastases is not achievable. The dilemma will be the choice between two detrimental therapeutic approaches: aspecific inhibition of the complete, antitumor and antipathogen neutrophil compartment with a main infection risk or ignoring neutrophils as targets in the TME as alternative ‘better’ therapies can be applied.

## Concluding remarks: ‘Confused’ neutrophils as targets in cancer therapy

Neutrophils are incredibly robust cells [15]. This is the consequence of the fight against and adaptation to ever-changing pathogens that try to manipulate this immune effector cell to evade killing by these cells. This has led to the fact that neutrophils are very difficult to manipulate via therapeutic strategies. In fact, no good direct antineutrophil therapeutics are available, and most powerful anti-inflammatory drugs are notoriously ineffective for the direct inhibition of neutrophils. This robustness, together with their plasticity in response to tissue cues, makes this cell type very difficult to manipulate/control. This issue holds true for both proinflammatory diseases and the pro- and antitumorigenic behavior of neutrophils in the TME. Therefore, the situation is best described by a type of confusion by which beneficial cytotoxic mechanisms are directed to tissues rather than pathogens and by which (antitumor) immunity is suppressed rather than activated. Would this mean that neutrophils are not worthwhile pursuing in anticancer therapies? The odds seem to favor this hypothesis, as neutrophils are difficult to change. However, little if anything is known regarding the underlying mechanisms of plasticity/confusion. This might be an opportunity, as differentiation decisions are most likely well controlled by, e.g., transcription factors [278, 279], bacterial products [280] and epigenetic mechanisms, and this control may occur mainly in the TME. If this is true, therapy development should focus on mechanisms of plasticity to allow for the inhibition of protumorigenic neutrophils but keep neutrophils in their normal antimicroorganism and antitumor phenotype, allowing protective immunity to take place. However, also an alternative approach deserves attention that is based on the hypothesis that ‘arming’ neutrophils in vivo with antitumor antibodies outweighs the risk of immune suppression by TANs. As it is not clear whether a balanced immune system is at all able to cope with tumors, one must choose one strategy over the other.
